# Right ventricular function–pulmonary pressure interaction as a predictor of mortality in tricuspid regurgitation

**DOI:** 10.1007/s10554-026-03753-2

**Published:** 2026-06-15

**Authors:** Yogev Peri, Yan Topilsky, Mohammad Alnees, Haitham Abu Khadija

**Affiliations:** 1 Heart Center, Kaplan Medical Center, Affiliated with the Hebrew University, Rehovot, Jerusalem, Israel; 2 Heart Center, Sourasky Medical Center, Sackler Faculty of Medicine, Tel Aviv University, Tel Aviv, Israel; 3 Postgraduate Medical Education, Global Clinical Scholar Research Training Program, Harvard Medical School, Boston, USA

**Keywords:** Tricuspid regurgitation, Right ventricular–pulmonary arterial coupling, TAPSE/PASP ratio, Right ventricular function, Pulmonary hypertension, Echocardiography, Mortality, Heart failure, Prognosis, Transcatheter tricuspid intervention

## Abstract

Right ventricular–pulmonary arterial (RV–PA) coupling reflects the ability of the right ventricle to adapt contractile performance to pulmonary afterload. Although TAPSE/PASP has been associated with outcomes across several cardiovascular conditions, its prognostic role in heterogeneous tricuspid regurgitation (TR) populations remains incompletely defined. To evaluate the association between RV–PA coupling, assessed by TAPSE/PASP, and mortality in patients with TR, and to explore whether a composite index integrating longitudinal and areal right ventricular function, (TAPSE × FAC)/PASP, provides incremental prognostic information. In this retrospective cohort study, 247 patients with any grade of TR and available echocardiographic measurements of TAPSE and PASP were included. RV–PA coupling was assessed using TAPSE/PASP, and patients were stratified according to the cohort median. A composite index, (TAPSE × FAC)/PASP, was also evaluated. The primary outcome was all-cause mortality. Cox proportional hazards regression and receiver-operating characteristic analysis were used to assess associations with mortality and discriminatory performance. A pre-specified subgroup analysis was performed in patients with moderate-to-severe TR, defined as EROA ≥ 0.30 cm². Among 247 patients, the median age was 80 years and 61% were female. Reduced TAPSE/PASP was associated with higher 1-year mortality compared with preserved TAPSE/PASP (41.9% vs. 16.3%, *p* < 0.001) and remained independently associated with mortality after multivariable adjustment (HR 2.15, 95% CI 1.35–3.43, *p* = 0.001). In the moderate-to-severe TR subgroup (*n* = 88), TAPSE/PASP retained prognostic significance (HR 2.74, 95% CI 1.42–5.28, *p* = 0.003). The composite index showed a modest improvement in discrimination compared with TAPSE/PASP alone (AUC 0.691 vs. 0.669). In this retrospective TR cohort, reduced TAPSE/PASP was independently associated with all-cause mortality, with consistent findings in patients with moderate-to-severe TR. The composite (TAPSE × FAC)/PASP index provided only modest incremental discrimination and should be considered exploratory and hypothesis-generating pending external validation.

**Graphical abstract:**

**Figure:**
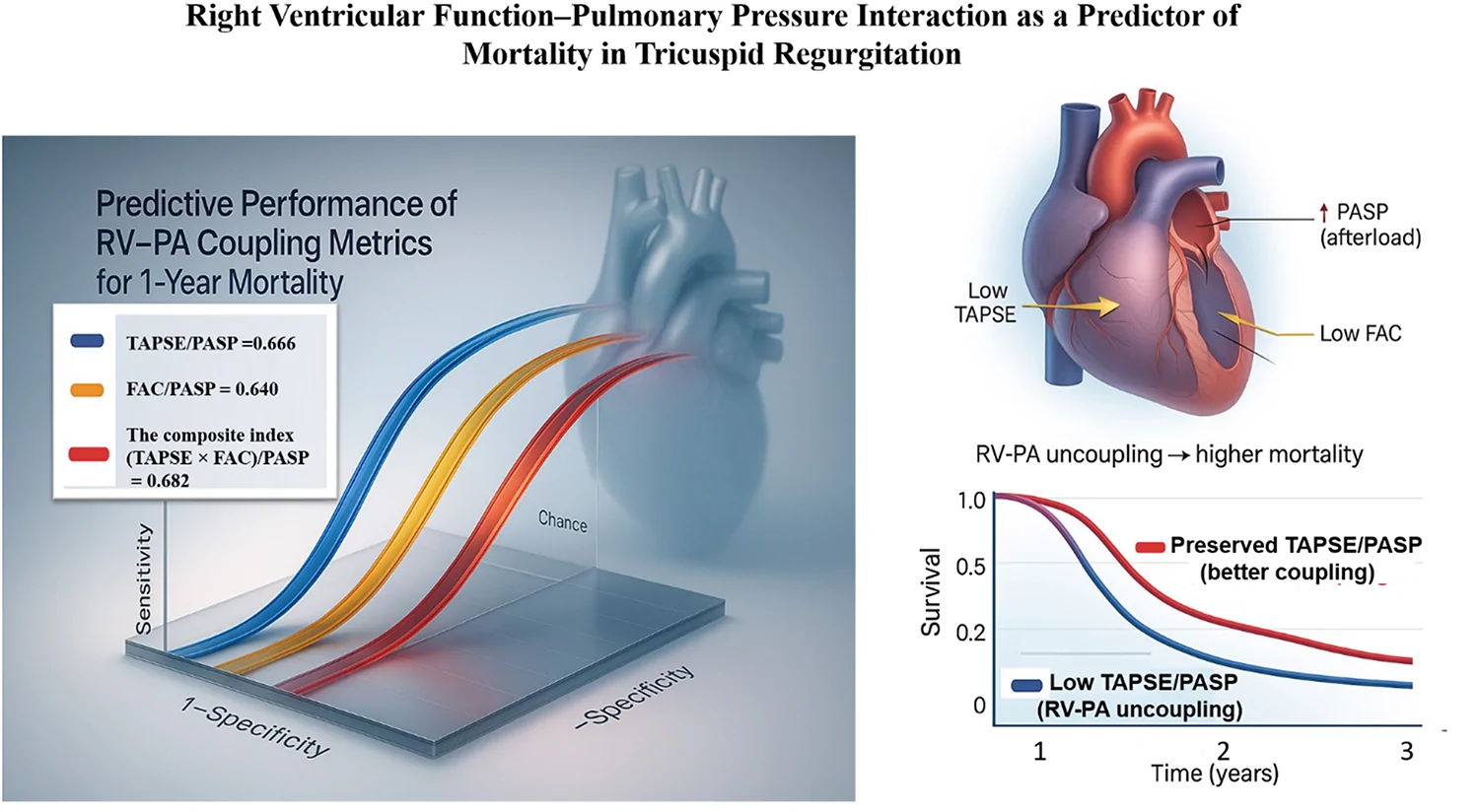

## Introduction

Tricuspid regurgitation (TR) is a common valvular disorder associated with significant morbidity and mortality. Clinically Significant TR (moderate or greater) is estimated to affect about 0.5% of the general population, with prevalence rising sharply in older adults. In fact, among individuals over 75 years old, roughly 1 in 25 has moderate or severe TR [1], making TR as prevalent in the elderly as aortic stenosis. Severe TR in particular portends a poor prognosis. Prior studies have shown a stepwise increase in mortality risk with increasing TR severity [2]. For example, in a large cohort, one-year survival declined from ~ 92% in patients without TR to ~ 64% in those with severe TR [3]. A 2019 meta-analysis confirmed that moderate or severe TR confers approximately a two-fold higher risk of all-cause death compared to none or mild TR, even after adjusting for pulmonary hypertension and right ventricular (RV) dysfunction [2].

The pathophysiologic connection between TR and mortality is inherently multifaceted. Most TR cases are secondary — arising from left-sided heart disease, atrial fibrillation, or pulmonary hypertension — which subjects the RV to chronic volume/pressure overload, annular dilation, and progressive dysfunction. The regurgitant flow burdens both the RV and the downstream venous system, ultimately leading to congestion and compromised forward output. In cohorts of advanced heart failure, severe TR is well-documented to carry a grim prognosis, with one-year mortality rates often approaching 30–40% [3]. Even in patients with preserved left ventricular function, moderate-to-severe TR independently predicts increased mortality and heart failure hospitalizations [1]. Consequently, TR should no longer be regarded as a benign bystander but rather as an active participant in disease progression [4].

A pivotal mediator of outcome in TR is right ventricular–pulmonary arterial (RV–PA) coupling, which describes how effectively the RV adjusts its contractile output to the pressure load imposed by the pulmonary circulation. The gold standard measurement, Ees/Ea (end-systolic to arterial elastance), requires invasive pressure–volume loop recordings, making it impractical for routine use [5]. Therefore, the echocardiographic TAPSE/PASP ratio has gained traction as a noninvasive surrogate [6, 7]. In a recent systematic review and meta-analysis, Anastasiou et al. summarized observational evidence across heart failure cohorts and confirmed that lower TAPSE/PASP is independently associated with increased mortality [8]. The ratio captures longitudinal RV shortening normalized to afterload, so a low value signifies RV–PA uncoupling, where reserve contractility is exhausted. However, despite robust data in heart failure and pulmonary hypertension (with TAPSE/PASP thresholds near ~ 0.35–0.40 mm/mm Hg linked to elevated risk), its prognostic role specifically in TR — a setting of concomitant pressure and volume overload — remains underexplored [5, 6, 8, 9]. Integrating TAPSE/PASP, or augmenting it with composite indices such as Right Ventricular Fractional Area Change (RV FAC), may therefore provide deeper insight for risk stratification in TR beyond relying solely on TR severity or RV function in isolation.

This study introduces a new parameter of RV–PA coupling that combines Tricuspid Annular Plane Systolic Excursion (TAPSE), Pulmonary Artery Systolic Pressure (PAPs), and Right Ventricular Fractional Area Change (RV FAC) to create TAPSE×FAC/PASP. We suggest that this composite measure may provide stronger prognostic value for predicting mortality in patients with all-cause severe TR.

## Methods

### Study population

**Fig. 1:**
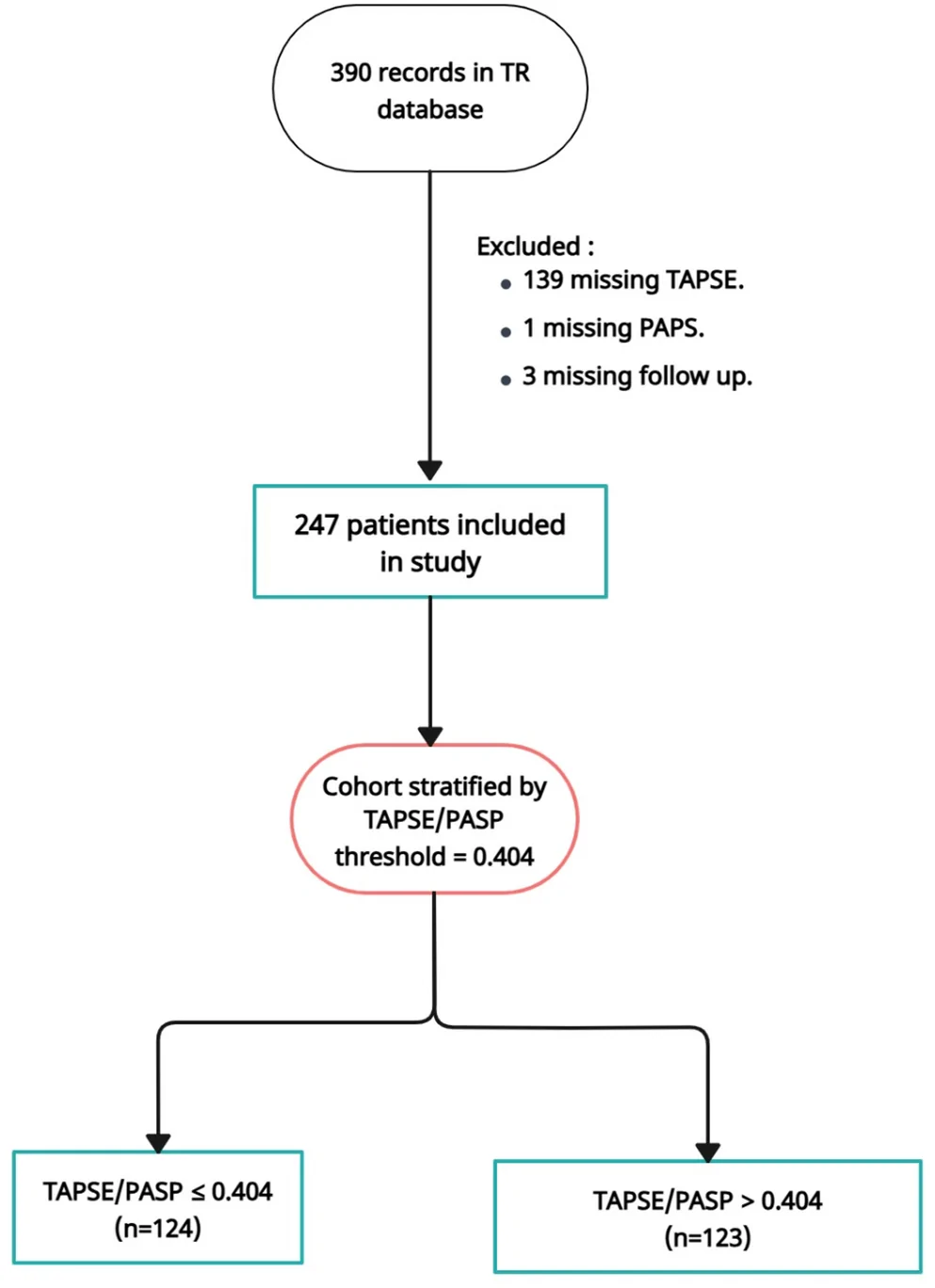
Study population flow diagram

We conducted a retrospective cohort study of patients with quantitatively assessed TR identified from an institutional echocardiography database at Tel Aviv Sourasky Medical Center, a tertiary heart center. Consecutive patients who underwent transthoracic echocardiography between January 2012 and December 2016 and were found to have any degree of TR were screened. From 390 initial patient records in the “all-cause TR” database, we included those with a complete echocardiographic assessment of RV function and estimated pulmonary pressures. Specifically, inclusion required that the study echocardiogram reported values for: [1] tricuspid annular plane systolic excursion (TAPSE), and [2] pulmonary artery systolic pressure (PASP, derived from TR jet velocity). We excluded patients if TAPSE was not measured (*n* = 139) or PASP could not be estimated (*n* = 1) from the echocardiogram, or if key clinical data or follow-up information were unavailable (*n* = 3). The final analysis cohort consisted of 247 patients with TR and complete data (Fig. 1).

The study population included patients with any grade of tricuspid regurgitation. Given the heterogeneity of TR severity and mechanisms, a pre-specified subgroup analysis was performed in patients with moderate-to-severe TR, defined as EROA ≥ 0.30 cm², to evaluate the robustness of the findings in a clinically relevant higher-risk population. Detailed etiologic subclassification of TR, including atrial functional, ventricular functional, device-related, and mixed mechanisms, was not systematically available and was therefore not incorporated into subgroup analyses.

### Clinical and echocardiographic

Baseline clinical characteristics, including age, sex, and relevant comorbid conditions (e.g., history of atrial fibrillation, left ventricular ejection fraction [LVEF] < 50%, and presence of pulmonary hypertension), were recorded. Standard transthoracic echocardiographic parameters were obtained from the index echocardiogram reports. TR severity was quantified according to current guideline recommendations using an integrative approach. Where available, the effective regurgitant orifice area (EROA) of the TR jet was recorded as a quantitative measure of TR severity [10]. In our laboratory, EROA was measured by the proximal isovelocity surface area (PISA) method for significant TR. Based on EROA and color Doppler jet parameters, TR severity was categorized as mild, moderate, severe, or torrential (massive). Severe TR was generally defined by EROA ≥ 0.40 cm² (or a vena contracta width ≥ 7 mm), while torrential TR was defined by an extremely large regurgitant orifice (> 0.70 cm²) consistent with recent recommendations for grading very severe TR. Right atrial pressure was estimated from inferior vena cava size and collapsibility on echocardiography, and this was used in calculating PASP from the TR jet peak velocity (using the modified Bernoulli equation). Pulmonary hypertension (PH) on echo was defined as an elevated PASP (typically PASP > 50 mm Hg, corresponding to at least moderate PH). Left ventricular ejection fraction was measured by Simpson’s biplane method or visually estimated if poor image quality.

Key RV functional parameters were collected. TAPSE was measured on M-mode from the apical 4 4-chamber view as the longitudinal excursion of the lateral tricuspid annulus from end-diastole to end-systole (in millimeters). RV fractional area change (FAC) was calculated as.

$$\text {RV FAC (\%) }= [(\text {RVEDA} - \mathrm{RVESA)} / \mathrm{RVEDA}] \times100.$$

$$\text {\mathbf{RVEDA}} = \text {Right Ventricular End-Diastolic Area}$$

$$\text {\mathbf{RVESA}} = \text {Right Ventricular End-Systolic Area}$$

obtained from planimetry of the RV cavity in the apical 4-chamber view. The TAPSE/PASP ratio was computed for each patient by dividing TAPSE (in mm) by PASP (in mmHg), yielding a coupling ratio in units of mm/mmHg. A higher TAPSE/PASP indicates better RV–PA coupling (relatively preserved RV function for the given afterload).

For certain analyses, patients were stratified into two groups based on RV–PA coupling status: an “uncoupled” group with TAPSE/PASP at or below the cohort median, and a “preserved coupling” group with TAPSE/PASP above the median. Similarly, we defined a novel composite index of RV–PA coupling, TAPSE×FAC/PASP, calculated by multiplying TAPSE (mm) by RV FAC (%) and dividing by PASP (mm Hg). This composite index has units of (mm×%) per mm Hg, but is interpreted as an integrated marker of RV contractile function adjusted for afterload. For comparison, we also computed a simple FAC/PASP ratio (substituting FAC in place of TAPSE).

All echocardiographic parameters were obtained from clinical reports, and no systematic offline re-measurement was performed. Measurements were acquired as part of routine clinical practice by experienced operators.

The study was conducted under a waiver of informed consent due to its retrospective nature, and it was approved by the institution’s ethics committee.

### Outcome measures

The primary outcome was all-cause mortality at 1 year. Long-term survival up to 3 years was also examined. Vital status and dates of death were ascertained through review of medical records and national death registries. For patients who underwent any tricuspid valve intervention or left-heart surgery during follow-up, those events were noted, but patients remained in the analysis (outcomes were analyzed by intention-to-treat, given the focus on initial risk stratification). However, the cohort predominantly received medical therapy during the follow-up period, as transcatheter tricuspid interventions were not widely available in the study era.

### Statistical analysis

Continuous variables were expressed as mean ± standard deviation or median with interquartile range (IQR), depending on distribution, and categorical variables as counts and percentages. Baseline characteristics were compared between the RV–PA uncoupling (low TAPSE/PASP) vs. preserved coupling (high TAPSE/PASP) groups using appropriate tests: the Student’s t-test or Mann–Whitney U test for continuous measures, and the Chi-square test or Fisher’s exact test for categorical variables. The primary analysis evaluated the association of RV–PA coupling with mortality. Kaplan–Meier survival curves were constructed for patients with low versus high TAPSE/PASP, and compared using the log-rank test. One-year mortality rates were reported for each group. We also performed a subgroup Kaplan–Meier analysis in the subset of patients with at least moderate-to-severe TR, defined as EROA ≥ 0.30 cm², to assess coupling in a higher-TR-severity subgroup.

Cox proportional hazards regression was used to identify predictors of all-cause mortality. Univariable Cox models were first run for TAPSE, PASP, TAPSE/PASP, and TAPSE×FAC/PASP, as well as for clinical covariates (age, sex, comorbid conditions, LVEF, TR severity grade, right atrial pressure, creatinine, hemoglobin, etc.). Variables with a significant association (*p* < 0.10) on univariate analysis, along with clinically important covariates from prior studies [2], were entered into multivariable Cox models. Two multivariable models were constructed: one including TAPSE/PASP as the measure of RV–PA coupling, and another substituting the composite index (TAPSE × FAC/PASP) to see if the composite provided added prognostic value. Hazard ratios (HR) with 95% confidence intervals (CI) and p-values were reported. Proportional hazards assumptions were checked by examination of Schoenfeld residuals.

To compare the discrimination of different echocardiographic metrics for 1-year mortality, we generated receiver operating characteristic (ROC) curves and calculated the area under the curve (AUC) (c-statistic) for the following indices: TAPSE alone, PASP alone, TAPSE/PASP ratio, FAC, FAC/PASP, TR EROA, and the composite TAPSE×FAC/PASP. Pairwise comparisons of AUC were performed using DeLong’s test. We also identified the optimal cutoff value of TAPSE/PASP for predicting 1-year mortality, defined by the maximum Youden’s index on the ROC curve (sensitivity + specificity – 1). A similar analysis was done to explore a cutoff for the composite index. All tests were two-tailed, with *p* < 0.05 considered statistically significant. Analyses were performed using SPSS (version 25, IBM Corp.) and MedCalc (for ROC comparison).

## Results

Among 247 patients (median age 80 years; 61% female), 124 (50%) had reduced RV–PA coupling (TAPSE/PASP ≤ 0.404). Baseline demographic, clinical, laboratory, and echocardiographic characteristics are summarized in Table 1. Pulmonary hypertension (defined as at least moderate) was more frequent (25.0% vs. 10.6%; *p* = 0.005), with higher right atrial pressure (16.3 ± 4.7 mm Hg vs. 12.2 ± 6.0 mm Hg; *p* < 0.001), higher serum creatinine (1.35 [1.03–1.80] vs. 1.07 [0.83–1.50] mg/dL; *p* < 0.001), and lower hemoglobin (10.99 ± 1.76 vs. 11.62 ± 2.07 g/dL; *p* = 0.017). Echocardiographically, they showed higher PASP (55 [45–66] vs. 34 [27–39] mm Hg; *p* < 0.001), lower TAPSE (14 [11–17] vs. 20 [17–24] mm; *p* < 0.001), and lower RV FAC (33 [23–44] vs. 42 [32–52] %; *p* < 0.001). Other baseline features, including LV size and left-sided valve disease, were comparable between groups.

**Table 1:** Baseline characteristics

		Overall	TAPSE/PAPS > 0.404	TAPSE/PAPS ≤ 0.404	*p*-value
		*n* = 247	*n* = 123	*n* = 124	
Age	*median (IQR)*	80 (69.5;80)	80 (69.5;84)	79.5 (70.5;85.25)	0.449
Male sex	*n (%)*	96 (38.9)	48 (39.0)	48 (38.7)	1
HTN	*n (%)*	150 (60.7)	70 (56.9)	80 (64.5)	0.274
DM2	*n (%)*	70 (28.3)	29 (23.6)	41 (33.1)	0.13
HLP	*n (%)*	100 (40.5)	52 (42.3)	48 (38.7)	0.659
Afib	*n (%)*	139 (56.3)	72 (58.5)	67 (54.0)	0.558
PHT	*n (%)*	44 (17.8)	13 (10.6)	31 (25.0)	**0.005**
IHD	*n (%)*	96 (38.9)	47 (38.2)	49 (39.5)	0.936
Previous MI	*n (%)*	41 (16.6)	17 (13.8)	24 (19.4)	0.318
Creatinine (mg/dl)	*median (IQR)*	1.215 (0.9125;1.6)	1.07 (0.83;1.5)	1.35 (1.025;1.795)	**< 0.001**
Hemoglobin (g/dl)	*mean (SD)*	11.29 (1.93)	11.62 (2.07)	10.99 (1.76)	**0.017**
RAP (mmHg)	*mean (SD)*	14.28 (5.74)	12.23 (6.02)	16.30 (4.65)	**< 0.001**
TR_ERO (cm²)	*median (IQR)*	0.30 (0.21;0.43)	0.33 (0.21;0.44)	0.28 (0.21;0.43)	0.131
TR_Reg_Vol (ml)	*median (IQR)*	28.90 (21.15;40.60)	27.7 (19.35;39.9)	31.25 (23.275;41.15)	0.124
PAPS (mmHg)	*median (IQR)*	41 (33;55)	34 (27;39)	55 (45;66)	**< 0.001**
TAPSE (mm)	*median (IQR)*	17 (13;21)	20 (17;24)	14 (11;17)	**< 0.001**
RV_FAC (%)	*median (IQR)*	38 (26;48)	42 (32;52)	33 (23;44)	**< 0.001**
LVEDD (mm)	*median (IQR)*	47.5 (42;54.25)	47 (43;53.5)	48 (41;56)	0.803
LVESD (mm)	*median (IQR)*	31 (26;40.25)	30 (26;38)	32 (25.75;44)	0.422
AS (moderate to severe)	*n (%)*	36 (14.6)	15 (12.2)	21 (16.9)	0.381
AR (severe)	*n (%)*	4 (1.6)	4 (3.3)	0 (0.0)	0.128
MS (moderate to severe)	*n (%)*	7 (2.8)	2 (1.6)	5 (4.0)	0.45
MR severity					
None		179 (74.3)	97 (79.5)	82 (68.9)	
Moderate		35 (14.5)	11 (9.0)	24 (20.2)	0.083
Moderate-to-severe		13 (5.4)	6 (4.9)	7 (5.9)	
Severe		12 (5.0)	6 (4.9)	6 (5.0)	
Prosthetic MR		2 (0.8)	2 (1.6)	0 (0.0)	N/A
IVSd (mm)	*mean (SD)*	11.36 (2.38)	10.77 (2.19)	11.94 (2.42)	**< 0.001**

*Abbreviations*: HTN = Hypertension; DM2 = Type 2 Diabetes Mellitus; HLP = Hyperlipidemia; Afib = Atrial Fibrillation; PHT = Pulmonary Hypertension; IHD = Ischemic Heart Disease; MI = Myocardial Infarction; RAP = Right Atrial Pressure; TR_ERO = Tricuspid Regurgitation Effective Regurgitant Orifice Area; TR_Reg_Vol = Tricuspid Regurgitant Volume; PAPS = Pulmonary Artery Systolic Pressure; TAPSE = Tricuspid Annular Plane Systolic Excursion; FAC_RV = Right Ventricular Fractional Area Change; LVDd = Left Ventricular End-Diastolic Diameter; LVDs = Left Ventricular End-Systolic Diameter; AS = Aortic Stenosis; AR = Aortic Regurgitation; MS = Mitral Stenosis; MR = Mitral Regurgitation; IVSd = Interventricular Septal Diameter. Data are presented as median (interquartile range) or mean ± standard deviation for continuous variables and as n (%) for categorical variables. Significant p-value =< 0.05 in "Bold"

The overall cohort included patients with varying degrees of TR severity, with a median EROA of 0.21 cm², indicating that a substantial proportion of patients had mild TR.

## Discussion

In this study, we evaluated the prognostic role of right ventricular–pulmonary arterial (RV–PA) coupling, quantified by the ratio of tricuspid annular plane systolic excursion (TAPSE) to pulmonary artery systolic pressure (PASP), in patients with tricuspid regurgitation (TR). Lower TAPSE/PASP values were associated with higher 1-year all-cause mortality, and this association persisted after adjustment for demographic and clinical covariates, supporting TAPSE/PASP as a clinically relevant marker of adverse outcome in this population. In the pre-specified subgroup of patients with moderate-to-severe TR (EROA ≥ 0.30 cm²), reduced TAPSE/PASP remained associated with mortality, suggesting that RV–PA coupling may provide prognostic information beyond TR severity alone. The exploratory composite index (TAPSE × FAC)/PASP demonstrated modestly higher discriminatory performance than TAPSE/PASP alone, suggesting that integrative measures incorporating both longitudinal and areal RV systolic function adjusted for afterload may warrant further investigation.

Building on contemporary guidance that recommends a multiparametric appraisal of RV systolic function, rather than reliance on any single metric, we framed our analysis to juxtapose TAPSE with FAC and then embed both within load-adjusted indices. Recent ASE right-heart documents and expert statements emphasize combining TAPSE, FAC, tissue-Doppler S′, and RV free-wall strain to capture the complex geometry and load-sensitivity of the RV. Against this background, **TAPSE** offers simplicity and strong feasibility, but it is angle- and load-dependent and predominantly interrogates basal longitudinal shortening. **FAC**, by contrast, provides a more global 2-D view (longitudinal + radial components) and shows stronger correlation with CMR-derived RVEF—albeit with higher image-quality demands and meticulous border tracing. Accordingly, and to align physiologic measurement with afterload, we prospectively specified analyses that normalize RV systolic performance to pulmonary pressure. Operationally, we defined TAPSE/PASP, and—motivated by the complementary information content of FAC—tested a composite (TAPSE×FAC)/PASP that integrates longitudinal and areal shortening per unit afterload. The measurement definitions and analytic plan are detailed in the Methods. In our cohort, coupling-based indices discriminated risk better than single-parameter measures: TAPSE/PASP outperformed FAC/PASP and TR EROA for 1-year mortality (AUC 0.666), while (TAPSE×FAC)/PASP provided a modest incremental gain (AUC 0.682). These findings persisted in the severe-TR subgroup and are congruent with broader evidence linking RV–PA coupling (often operationalized as TAPSE/PASP) to outcomes across heart-failure phenotypes.

These findings underscore the significance of evaluating RV–PA coupling in patients with TR, a population frequently characterized by intricate hemodynamic interactions between the right-sided chambers and the pulmonary circulation. Traditional measures, such as TAPSE or PASP, in isolation provide valuable information but may not fully capture the integrated, load-adapted performance of the right ventricle. By combining these parameters, TAPSE/PASP offers a load-adjusted index of RV function that is both easily obtainable and clinically meaningful.

In the context of TR, assessing RV–PA coupling may be particularly valuable. Patients with significant TR often have a complex interplay between right-sided volume overload and pressure overload. TAPSE or RV fractional area alone might not fully capture functional status if the afterload varies widely among patients. For example, a patient with moderate TR and moderate PH (moderately elevated PASP) may have the same TAPSE as another patient with severe TR but a normal PASP; the prognosis might differ because the first patient’s RV is facing higher afterload relative to its contractility. TAPSE/PASP offers a convenient and non-invasive way to gauge this balance. It can be readily calculated from a standard echo, requiring no additional imaging beyond what is already done for TR assessment (TAPSE measurement and TR velocity for PASP). Given our findings, incorporating TAPSE/PASP into routine TR evaluation could help identify high-risk individuals. Patients with low TAPSE/PASP (uncoupling) might warrant closer monitoring, more aggressive heart failure therapy (to reduce afterload and support RV function), and consideration for earlier intervention on the tricuspid valve. This dovetails with emerging paradigms that TR should be intervened upon before irreversible RV failure ensues [1]. Indeed, uncoupling could serve as a red flag that the window for safe intervention may be narrowing.

Moreover, the strong association between low TAPSE/PASP and mortality suggests its incorporation into risk scores. Recently, a TRI-SCORE (tricuspid regurgitation) risk score has been proposed for surgical candidates, taking into account markers such as liver function, NYHA class, and TAPSE < 17 mm, among others. Given our findings, TAPSE/PASP could enhance risk stratification schemes by adding the dimension of afterload adjustment. It is conceivable that future risk models or guidelines for TR will include RV–PA coupling metrics to better stratify who is “too sick” vs. “just in time” for an intervention. In the meantime, clinicians should be aware that a patient with moderate TR and very low TAPSE/PASP may actually be at higher risk than one with severe TR and preserved coupling. The former should not be falsely reassured by the TR grade alone.

While many prior studies have documented the prognostic importance of TR severity and RV dysfunction in general, few have specifically examined noninvasive RV–PA coupling in a pure TR population [2, 3, 11, 12]. Our results align with the broader heart failure literature, which positions TAPSE/PASP as a robust predictor of outcomes. For example, Guazzi et al. demonstrated that TAPSE/PASP stratified mortality risk in HFpEF patients, effectively identifying those with underlying pulmonary vascular disease and RV dysfunction [12]. In secondary (functional) TR, which constitutes the majority of our cohort, recent analyses have likewise pointed to RV dysfunction and pulmonary pressure as critical drivers of prognosis [11]. Wang et al. (2019) reported that even after accounting for PASP and RV function, moderate/severe TR independently doubled mortality risk [2], implying that TR adds load that pushes marginal RVs over the edge. Our findings refine this concept: it is the failure of the RV to cope with that added load – manifest as RV–PA uncoupling – that most directly links to mortality. More studies in secondary TR have emphasized the central role of RV dysfunction in driving adverse outcomes, independent of TR severity itself [1].

In the context of interventions, our data complement findings from the TriValve registry (TTVR patients) [13] and other studies [14, 15]. For instance, in a cohort of patients undergoing MitraClip for mitral regurgitation, coexistent TR was a predictor of worse outcomes, and RV–PA coupling was noted to be an important determinant of post-procedural prognosis [14]. This underlines that regardless of whether patients undergo intervention or not, RV–PA uncoupling identifies a sicker phenotype. Some investigators have explored alternative ways to measure coupling, such as using RV free-wall longitudinal strain over PASP (RV GLS/PASP). In acute heart failure, an RV GLS/PASP ratio ≤ 0.32 (strain in absolute value divided by PASP) was associated with higher mortality, similar in spirit to TAPSE/PASP [15]. Those advanced measures (strain-based) may be more sensitive to subtle dysfunction, but TAPSE has the advantage of simplicity and availability. Our introduction of TAPSE×FAC/PASP is conceptually akin to combining multiple RV metrics with afterload. Prior studies did not use this exact composite; however, the idea is supported by the understanding that RV performance is multidimensional, and no single metric is perfect [16, 17]. The slight improvement in AUC with our composite index hints that a more nuanced assessment of RV function (e.g., incorporating 3D RV ejection fraction or strain) could further improve prognostication in TR. Future research could compare TAPSE/PASP with RV strain/PASP or 3D-derived metrics in TR populations.

### Limitations and future directions

Several limitations should be acknowledged. First, this was a retrospective, single-center study, which introduces the possibility of selection bias and residual confounding. Second, the study population included patients with varying degrees of tricuspid regurgitation severity, and a substantial proportion had mild TR, which may limit the clinical applicability of the findings to patients with clinically significant TR. Third, detailed etiologic subclassification of TR was not systematically available. Contemporary TR assessment increasingly recognizes the importance of distinguishing atrial functional TR, ventricular functional TR, device-related TR, and mixed mechanisms, as these entities may have distinct mechanistic, prognostic, and therapeutic implications. Because the present study was based on retrospective review of clinically reported echocardiographic data, systematic adjudication of TR mechanism, annular remodeling pattern, device-lead interaction, and right atrial or right ventricular remodeling was not consistently feasible. Fourth, echocardiographic parameters were derived from clinical reports without systematic offline re-measurement, and no reproducibility analysis was performed.

Fifth, TAPSE/PASP and related indices are load-dependent and rely on Doppler-derived estimates of PASP. PASP estimation from the tricuspid regurgitant jet may be less accurate in patients with severe or torrential TR, particularly when the Doppler envelope is incomplete or when right atrial and right ventricular pressures rapidly equalize. Pulmonary valve Doppler-derived parameters, such as pulmonary acceleration time and related Doppler surrogates of pulmonary hypertension, were not systematically available in this retrospective dataset and therefore could not be incorporated. Sixth, valvular interventions during follow-up were not modeled as time-dependent covariates, which may have influenced outcome assessment. Finally, the proposed composite index and its derived cut-off values were not externally validated and should therefore be considered exploratory and hypothesis-generating.

Future prospective, multicenter studies are warranted to validate the prognostic role of RV–PA coupling in clinically relevant TR populations, particularly in patients with moderate-to-severe disease. Further work should evaluate RV–PA coupling within etiologically defined TR subgroups and determine whether alternative afterload markers, including pulmonary valve Doppler-derived indices, improve risk assessment when TR-derived PASP estimation is limited. Additional studies are also needed to determine whether integrating multiple RV functional parameters provides clinically meaningful incremental value beyond established indices such as TAPSE/PASP.

### Take-home message

In patients with tricuspid regurgitation, right ventricular–pulmonary arterial coupling, assessed by TAPSE/PASP, was independently associated with all-cause mortality. A threshold around 0.40 mm/mmHg identified patients at higher risk in this cohort, including after adjustment for clinical covariates. Incorporating TAPSE/PASP into comprehensive echocardiographic assessment may provide additional risk-stratification information beyond TR severity alone and may help identify patients who require closer follow-up and multidisciplinary evaluation.

## Conclusion

In this retrospective cohort, reduced RV–PA coupling, assessed by TAPSE/PASP, was independently associated with all-cause mortality in patients with tricuspid regurgitation, with consistent findings in the subgroup with moderate-to-severe disease. These findings support the potential role of TAPSE/PASP as a clinically relevant marker of risk in TR. The composite index (TAPSE × FAC)/PASP demonstrated only modest incremental improvement in discrimination and should be considered exploratory and hypothesis-generating pending external validation.

From a clinical perspective, TAPSE/PASP is a simple, noninvasive parameter that may provide additional insight into right ventricular adaptation to pulmonary afterload. Its integration into routine echocardiographic evaluation may improve risk stratification; however, prospective validation in larger, etiologically characterized TR cohorts is required before it can be used to guide clinical decision-making.

**Fig. 2:**
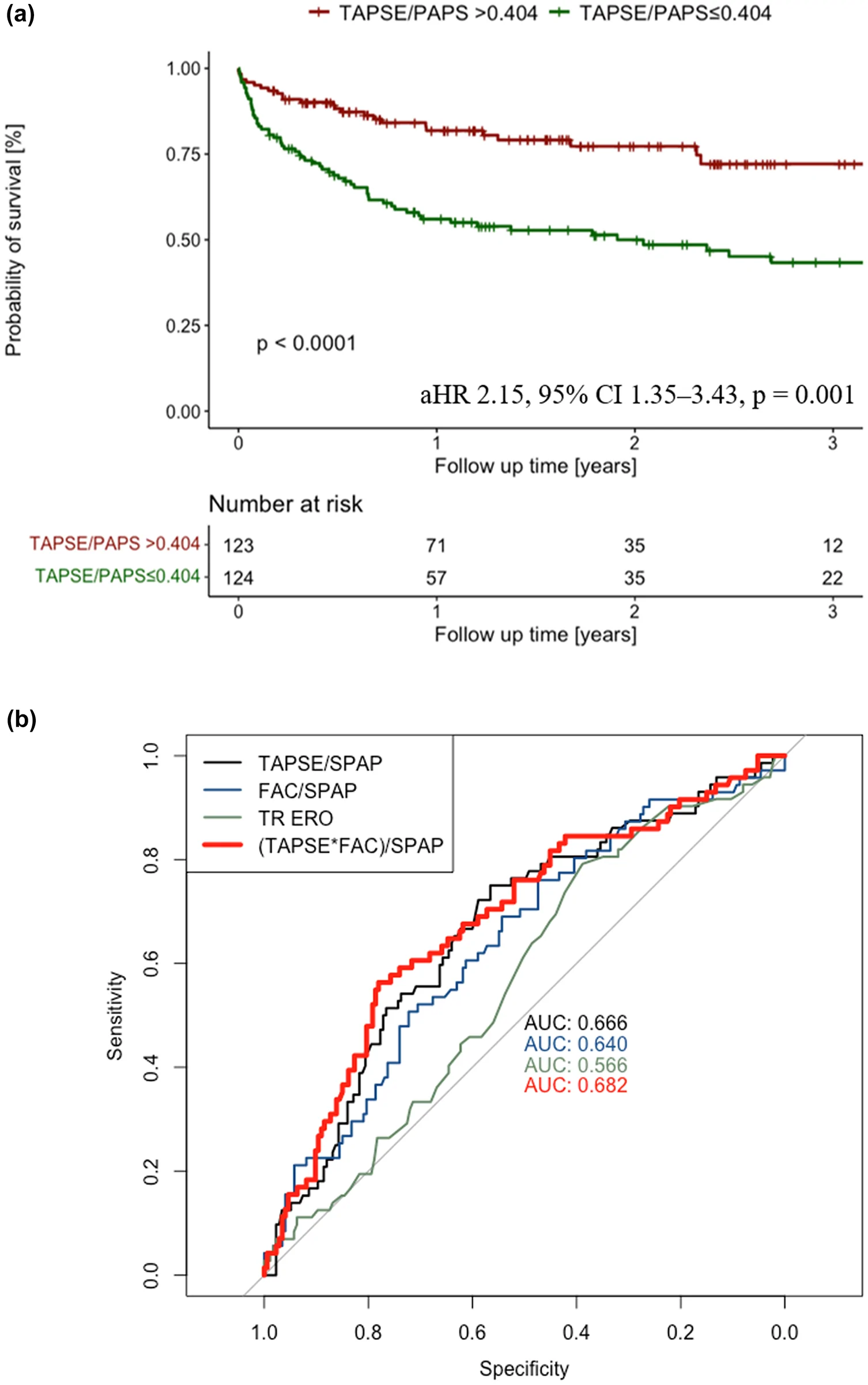
(**A**) All-cause mortality according to TAPSE/PASP ratio. (**B**) Receiver-Operating Characteristic (ROC) analysis for 1-year all-cause mortality

Kaplan–Meier survival analysis demonstrated a significantly higher all-cause mortality among patients with reduced RV–PA coupling (TAPSE/PASP ≤ 0.404) compared with those with preserved coupling (TAPSE/PASP > 0.404). The divergence in survival curves appeared early and persisted throughout the 3-year follow-up period (*log-rank p* < 0.0001). At 1 year, mortality was 41.9% in the low-coupling group versus 16.3% in the preserved-coupling group, confirming that impaired RV–PA coupling is a powerful predictor of adverse outcome in patients with tricuspid regurgitation, as illustrated in Fig. 2A. In multivariable Cox regression adjusting for age, sex, left ventricular function, and TR severity, TAPSE/PASP remained an independent predictor of mortality (adjusted hazard ratio [HR 2.15, 95% CI 1.35–3.43, *p* = 0.001).

Figure 2B illustrates the comparative performance of several echocardiographic parameters in predicting 1-year all-cause mortality. The TAPSE/PASP ratio demonstrated the strongest prognostic discrimination (AUC = 0.666), outperforming RV fractional area change–to–PASP (FAC/PASP, AUC = 0.640) and tricuspid regurgitation effective regurgitant orifice area (TR ERO, AUC = 0.566). A novel composite index that integrates longitudinal and areal RV systolic function— **(TAPSE × FAC)/PASP**—showed a modest incremental improvement (AUC = 0.682), suggesting slightly enhanced predictive value compared with TAPSE/PASP alone. These results confirm that indices reflecting **RV–PA coupling** provide better mortality discrimination than isolated measures of TR severity

**Fig. 3:**
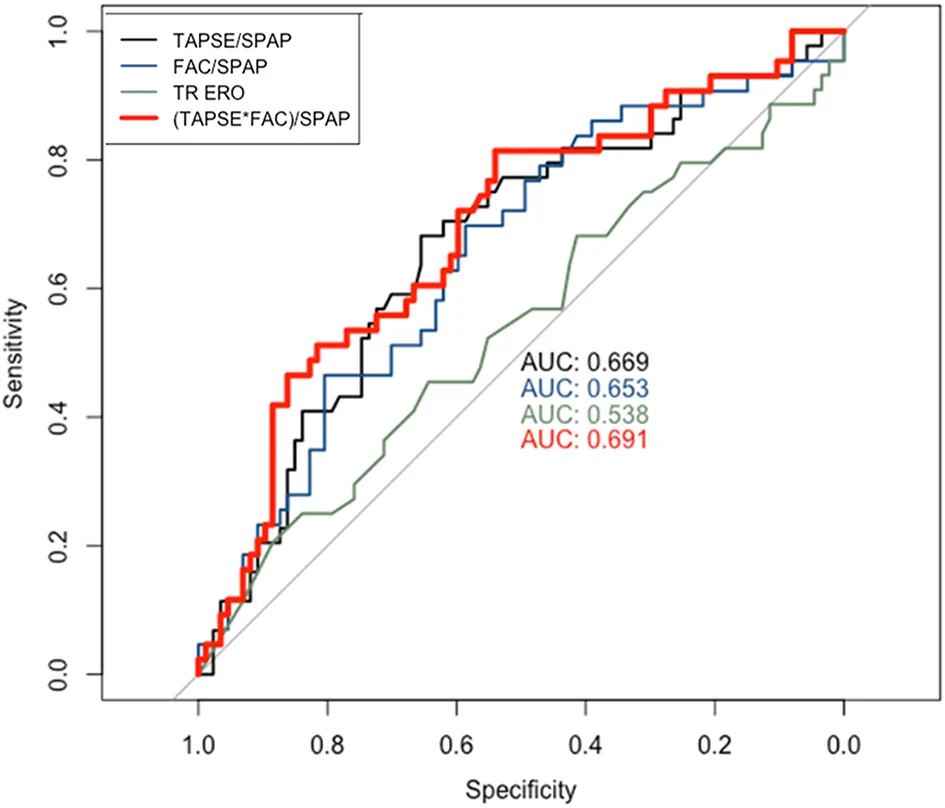
ROC analysis for prediction of 1-year all-cause mortality in severe TR subgroup

Figure 3 presents the receiver-operating characteristic (ROC) curves comparing echocardiographic indices for predicting 1-year all-cause mortality among patients with tricuspid regurgitation (EROA ≥ 0.30 cm²; *n* = 88). In this high-risk subgroup, TAPSE/PASP demonstrated strong prognostic discrimination (AUC = 0.669), outperforming FAC/PASP (AUC = 0.653) and TR effective regurgitant orifice area (EROA) (AUC = 0.538). The composite index (TAPSE × FAC)/PASP achieved the highest predictive accuracy (AUC = 0.691), reflecting modest but meaningful improvement over single-parameter metrics Consistent with these findings, one-year mortality was 38% in patients with low TAPSE/PASP compared with 14% in those with preserved coupling (log-rank *p* < 0.001). In multivariable Cox analysis, TAPSE/PASP independently predicted all-cause mortality (HR 2.74; 95% CI 1.42–5.28; *p* = 0.003), outperforming TAPSE or PASP considered individually

## Data Availability

The data that support the findings of this study are available from the corresponding author upon reasonable request. The data are not publicly available due to institutional and patient privacy restrictions.
